# Tuberculosis preventive treatment among individuals with inactive tuberculosis suggested by untreated radiographic abnormalities: a community-based randomized controlled trial

**DOI:** 10.1080/22221751.2023.2169195

**Published:** 2023-01-27

**Authors:** Haoran Zhang, Henan Xin, Ying Du, Xuefang Cao, Shouguo Pan, Jianmin Liu, Ling Guan, Fei Shen, Zisen Liu, Bin Zhang, Dakuan Wang, Boxuan Feng, Jiang Du, Xueling Guan, Yijun He, Yongpeng He, Zhanjiang Zhang, Jiaoxia Yan, Qi Jin, Lei Gao, for the LATENTTB TRIAL2-NSTM study team

**Affiliations:** a NHC Key Laboratory of Systems Biology of Pathogens, Institute of Pathogen Biology, and Center for Tuberculosis Research, Chinese Academy of Medical Sciences and Peking Union Medical College, Beijing, People’s Republic of China; b Center for Diseases Control and Prevention of Zhongmu County, Zhengzhou, People’s Republic of China; c The Sixth People’s Hospital of Zhengzhou, Zhengzhou, People’s Republic of China

**Keywords:** Latent tuberculosis infection, preventive treatment, radiographically inactive tuberculosis, fibrosis, randomized controlled trial

## Abstract

Epidemiological and interventional studies have been rarely conducted among those with positive interferon-γ release assay (IGRA) results and radiologically inactive tuberculosis (TB) lesions on chest radiograph. This study aimed to estimate the effectiveness and safety of a six-week twice-weekly regimen (rifapentine plus isoniazid) among this key population in rural China. First, chest digital radiography was conducted to screen individuals with inactive TB lesions. Then, the identified participants were further evaluated and eligible participants with IGRA-positive results were included in subsequent randomized controlled trial (RCT). Of 44,500 recruited residents, 2,988 presented with radiographically inactive TB among 43,670 with complete results of chest radiography and questionnaire, and 28.61% (855/2,988) tested IGRA positive. Subsequently, 677 eligible participants were included in this RCT (345 in the preventive treatment group and 332 in the untreated control group). The treatment completion rate was 80.00% (276/345), and 11.88% (41/345) participants reported side-effects including two cases of hepatotoxicity (0.58%, 2/345). In the intention-to-treat analysis, the cumulative incidence rate of microbiologically confirmed active TB during a two-year follow-up was 1.16 (95% confidence interval [CI]: 0.03–2.29) in the preventive treatment group and 1.51 (95% CI: 0.20–2.82) in the control group (*p *= .485). Subgroup analyses showed that the protective rates were 55.42% (95% CI: 10.33–93.07%) and 80.17% (95% CI: 25.36–97.96%) for participants with fibrosis and for those aged ≥60 years, respectively. The expected treatment effect was not observed for the six-week regimen in this study. Future studies with sufficient sample size are needed to verify our findings.

## Introduction

According to the estimation by the World Health Organization (WHO), about a quarter of the world’s population was infected with *Mycobacterium tuberculosis* (MTB), and approximately 5%–10% MTB infections might progress to active tuberculosis (TB) in their lifetimes [1,2]. Promoting the testing and treatment of latent tuberculosis infection (LTBI) among high-risk populations is a critical tool for the END TB. Obviously, population-based LTBI testing and treatment is infeasible, and given the vast pool of infections, strategies centred on high-risk groups will provide greater cost effectiveness. Identifying targets for preventive treatment, which could incur a great epidemiological value, needs to be firstly addressed when developing local guidelines for LTBI management.

The 2020 updated WHO guidelines recommended that individuals with a history of inactive TB or fibrotic lesions should not be a contraindication for preventive treatment, due to the increased risk of active disease progression [3]. Secondary preventive treatment has been practically recommended for individuals with abnormal chest radiographic findings suggestive of inactive TB (untreated or inadequate treated) in America and Canada due to the 6–19 times increased risk of developing active disease as compared to those with normal chest radiography [4,5]. Our previously prospective study consistently found that individuals with inactive TB suggested by untreated chest radiographic abnormalities showed an increased risk of developing active TB, this subgroup contributed about 30% of TB cases occurred in our study population in rural China [6,7]. However, little was known about the burden of this specific population and their TB infection status in China. Under this circumstance, we firstly conducted a population-based large-scale screening study to identify eligible participants among rural residents to establish baseline results for further interventions.

In addition, our previous studies have proved the efficacy of a six-week regimen (rifapentine plus isoniazid) as 69% in two years [8] and 61% in five years [9] among Chinese rural residents. Therefore, the primary aim of this subsequent randomized controlled trial (RCT) was to evaluate the safety and two-year protective effect of this short-course regimen for TB preventive treatment among individuals with inactive TB suggestive untreated by radiographic abnormalities. This study was expected to promote the exploration of suitable TB preventive treatment for this specific population in China and areas with high burden of inactive TB.

## Methods

### Trial design

**Figure 1.**
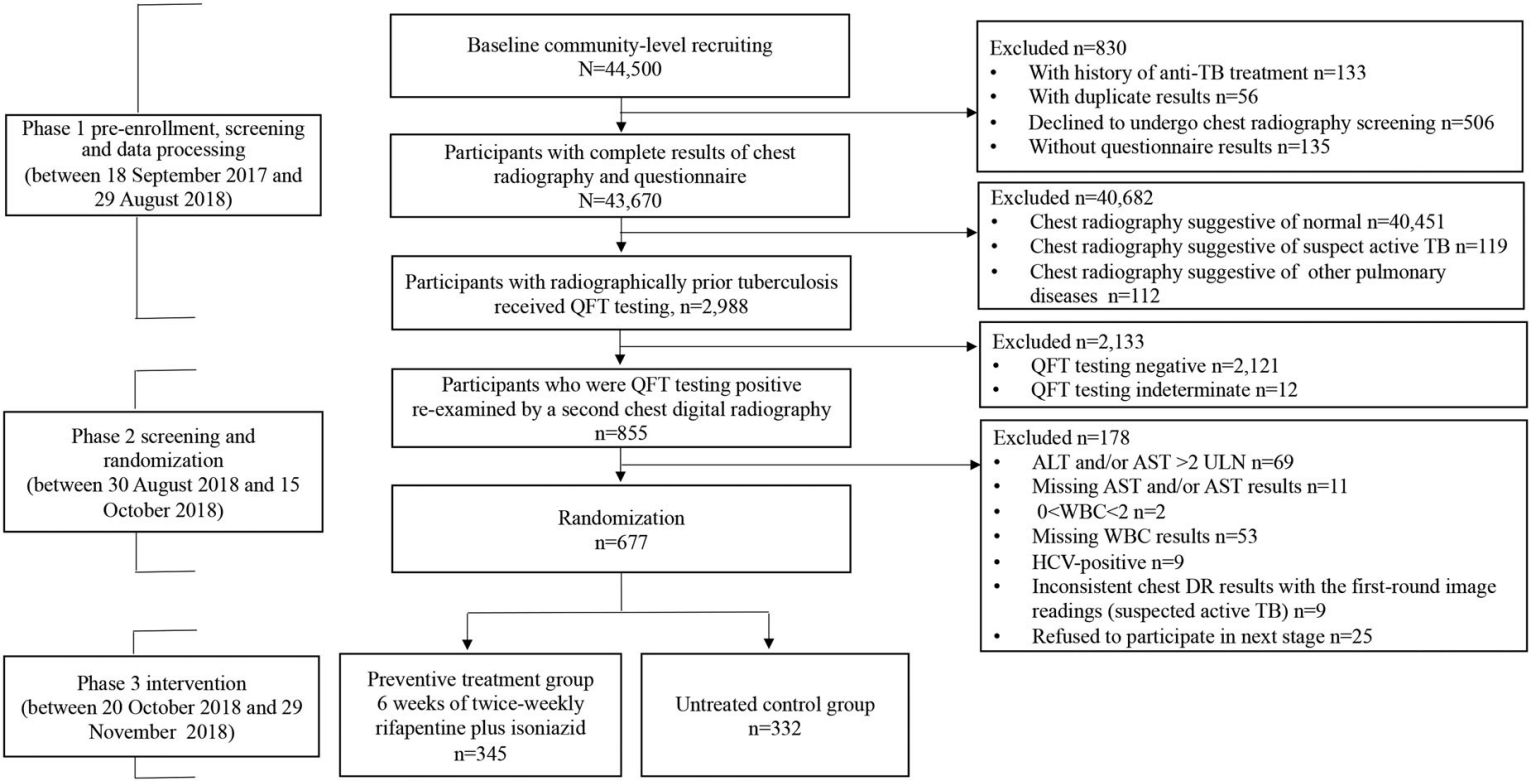
Flowchart of the participant enrolment. ALT, alanine aminotransferase; AST, aspartate aminotransferase; DR, digital radiography; HCV, hepatitis C virus; QFT, QuantiFERON-TB Gold In-Tube; RCT, randomized controlled trial; TB, tuberculosis; ULN, upper limit of normal; WBC, white blood cell. Of the 44,500 recruited rural residents, 43,670 participants aged 18–75 years had complete results of chest radiography and questionnaire, 40,682 were excluded because of normal chest radiographic findings (*n* = 40,451), suspect TB (*n* = 119) and other pulmonary diseases (*n* = 112). The rest 2,988 participants were identified with radiographically inactive TB lesions. Among them, 855 individuals were identified to be QFT-positive and 830 signed the informed consent form for the RCT. Finally, 677 eligible participants were included in the RCT and were randomly classified into two groups: 345 in the preventive treatment group and 332 in the control group without treatment.

The study, which conducted in rural communities in Zhongmu County with an average TB incidence of 57 per 100,000 for three consecutive years (2015–2017), consisted of two phases: a community-level screening study based on chest digital radiography (DR) investigations (two-round screening survey, see Figure 1) and an open-labelled two-arm RCT including a preventive treatment group and control group without intervention. The control group was settled without preventive treatment because there was currently no recommendation for this population in the guidelines of WHO or China [3,10]. The regimen was six weeks of twice-weekly rifapentine (at a maximum dose of 600 mg; at a dose calculated based on body weight, with incremental adjustment for participants who weigh ≤50 kg, for example, if a participant weighs 38–50 kg, the dose of drug he or she should take is 450 mg; and at a dose of 600 mg for those who weigh >50 kg) plus isoniazid (at a maximum dose of 600 mg; carried and rounded off at a dose of 15 mg/kg for those who weigh ≤50 kg, for example, if a participant weighs 43 kg, the dose of drug he or she should take is calculated as 600-[15 mg/kg*(50–43)] = 495, so 500 mg will be taken; and at a dose of 600 mg for those who weigh >50 kg).

This trial was registered at the Chinese Clinical Trial Registry (ChiCTR-1800018224). The protocol was approved by the ethics committees of the Institute of Pathogen Biology, Chinese Academy of Medical Sciences, Beijing, China (IPB-2018-03-12). Written informed consent was obtained from each study participant. Proswell Medical Company (Beijing, China) independently monitored the study during its implementation.

### Study participants and sample size calculation

For the enrolment screening, eligible participants were required to (1) be aged 18–75 years; (2) be local household registration of rural residents or continuous residence at the study site for at least six months [11] and have no migration plan in the next three years; (3) be self-committed to complete the entire study period; and (4) be willing to sign the informed consent form. Exclusion criteria were (1) residents with current active TB or suspected TB (suspected TB was defined by chest radiographic abnormalities that were suggestive of a probable active TB but without microbiologic evidence according to the National Health Industry Standard on Diagnosis for Pulmonary Tuberculosis [WS288-2017]) [12]; (2) residents with previous anti-TB treatment history confirmed by checking information recorded in the national Tuberculosis Information Management System (NTIMS); or (3) residents who were pregnant or expectant women.

The RCT inclusion criteria were (1) residents with radiographical lesions suggestive of inactive TB, which were adapted from the National Health Industry Standard on Classification of Tuberculosis (WS196-2017) [13], including fibrotic lesions, calcification, nodule, and pleural incrassation; and (2) QuantiFERON-TB Gold In-Tube (QFT, a commercial product of Interferon-γ release assay [IGRA]; Qiagen, Germantown, MD, USA) positive results (a cut-off value of ≥0.35 IU/mL recommended by the manufacturer). The participants would be excluded if they met one of the following exclusion criteria: (1) contraindication for rifapentine or isoniazid, (2) history of LTBI treatment with rifapentine persistently for >14 days or with isoniazid intermittently for >30 days in the past two years [14]; (3) serum alanine aminotransferase (ALT) and/or aspartate aminotransferase (AST) >2 times the upper limit of normal (ULN), and/or accompanied by liver impairment symptoms and signs; (4) white blood cell (WBC) count <2.0 × 10^9^ L^−1^; (5) positivity of antibody to hepatitis C virus (HCV) and/or positive for antibody to human immunodeficiency virus (HIV); (6) with renal insufficiency or degeneration; (7) addiction to drinking or actively using street drugs; (8) cancer; and (9) mental disorders or disability.

The sample size was calculated with the aim of reducing TB incidence in the intervention group by 70% in two years with a risk of active TB development of 2.6% in intervention group [9], and 229 participants per group would provide a power of 80% based on a two-sided alpha level of 0.05. Assuming 20% treatment discontinuation and 15% loss to a two-year follow-up, the final estimated sample size was 674 participants.

### Community-level screening and intervention participant recruitment

Between 18 September 2017 and 29 August 2018, a community-level baseline screening was initially conducted to identify participants with radiographically inactive TB using chest DR examination. To improve the accuracy of the radiographic diagnosis of inactive TB, two-round chest DR screening combined with MTB infection testing was conducted. In the first round, participants who were initially identified with radiographically inactive TB lesions by one radiologist would be introduced for QFT testing. Those with QFT-positive results were then invited for the second-round screening survey, in an interval of three to six months (between 30 August 2018 and 15 October 2018) to identify eligible participants for the RCT. Specifically, chest DR was repeated for QFT-positive participants with radiographically inactive TB lesions to exclude unstable lesions with progression. In this round of screening, a panel of radiologists, consisting of one local radiologist and one radiologist from provincial infectious diseases hospital, read each image together and provided a common report after discussion. If the participants were confirmed to have radiological findings suggestive of suspected TB, microbiological testing including sputum smear, culture and GeneXpert MTB/RIF assay (Cepheid, Sunnyvale, CA, USA) would be done to exclude currently active TB. TB diagnosis was made according to the National Health Industry Standard on Diagnosis for Pulmonary Tuberculosis (WS288-2017) [12]. The standard report form was shown in Supplementary Table S1. Additionally, each participant completed a standardized questionnaire to obtain sociodemographic information, as well as blood routine examination and biochemistry tests. After comprehensively considering the test results, eligible participants for the RCT were determined.

### Randomization

Eligible participants selected for the RCT were randomly classified into the preventive treatment group or control group by sex using computer-generated random numbers. All eligible participants were centrally allocated a sequential study identifier by the RCT statistician and then randomly assigned to one of the two groups. Participants, study team, and statistical analysts were not blinded to treatment assignment. However, the panel members for evaluation and diagnosis of TB were fully unaware of the trial-group assignments.

### Intervention implementation

At each village clinic visit, to prevent control contamination, the study drugs were dispensed and taken after meals under direct observation by trained village doctors. Participants had no access to prescription drugs by themselves. During the pre-specified six-week phase, participants in the preventive treatment group were allowed to take the medicine within three days in case of interruptions. However, for the last dose, considering the need for more physical examination and information collection, participants were allowed for seven days’ delay in order to improve the adherence [14]. The occurrence of adverse events (AEs), including gastrointestinal reactions, neurological symptoms, hypersensitivity or allergy, influenza-like symptoms and other systemic drug reactions, were recorded by the researchers on each visit before dispensing medicine every time. Participants were asked to return to the clinic if they felt unwell at any time throughout the treatment course. To closely monitor side-effects, scheduled clinical examinations and evaluations, including blood routine examination and blood biochemistry tests (test indicators included AST, ALT, total bilirubin, creatinine, and urea nitrogen, etc.), were conducted once every two weeks during the treatment period. Assessment of long-term side-effects occurred at three-month visit after the last medication.

### Follow-up survey and endpoints

All participants including untreated controls were followed up for 24 months. At quarter follow-up, participants were investigated of suspected symptoms through door-to-door or telephone survey. At annual follow-up, participants were invited for investigation on suspected symptoms and chest DR examination. Participants with evidence of suspected TB were transferred to the local Center for Diseases Control and Prevention for final diagnosis. Three sputum samples were collected from each participant for sputum smear, culture and GeneXpert MTB/RIF assay. On the basis of National Health Industry Standard on Diagnosis for Pulmonary Tuberculosis (WS288-2017) [12], individuals with positive results for culture and/or GeneXpert MTB/RIF assay, were diagnosed with microbiologically confirmed TB cases. Response to empiric TB treatment among participants suspected as having TB but negative to microbiological tests was defined as clinically diagnosed TB. Cases registered in NTIMS during the follow-up period were also included in the present study. In consideration of the suboptimal accuracy of clinical diagnosis in this special population, microbiologically confirmed TB was regarded as the primary study endpoint in order to more precisely evaluate the efficacy of the regimen. Furthermore, to rule out initial misclassification of co-prevalent cases, we excluded individuals diagnosed with TB during the first quarterly follow-up period from the analysis.

Treatment completion, permanent discontinuation, or death from any cause were also analysed. Treatment completion was defined as completion of ≥11 of 12 doses and was comprehensively assessed by pill counts and drug registration. AEs and study drug-related side-effects were graded by researchers according to the Common Terminology Criteria for Adverse Events (CTC-AE, version 4.0). The attribution of drug-related side-effects was co-determined by two external physicians using a scale ranging from unrelated to definitely related (Supplementary Table S2).

Temporary or permanent discontinuation was judged by physicians and principal investigators based on clinical evaluation. For permanently discontinued participants, follow-up lasted until the end of two-year follow-up.

### Statistical methods

Analyses were performed using SAS (SAS Institute, version 9.4). Between-group comparisons for categorical variables were estimated by Chi-squared test with a two-sided significance level of .05. The intention-to-treat (ITT) analysis included all participants enrolled in the two groups. The per-protocol analysis included participants who completed the study regimen. The cumulative incidence and incidence rate per 100 person-year with 95% confidence interval (CI) was calculated at 24 months. The differences in the incidence of active TB between the preventive treatment and untreated control groups were assessed using a one-sided significance level of .05. Due to lost to follow-up issues, Kaplan–Meier time-event analysis was used. Person-year for each participant was calculated based on the quarterly and annual follow-up records for 24 months after treatment. If the participants did not participate in the two-year survey, the duration for which they remained in the study was calculated based on the records of the last completed quarterly follow-up. If the participants had died of other causes, the person-years were calculated based on the records of time of death. If necessary, Cox proportional hazards regression models and an unconditional multiple logistic regression analysis would be performed to identify respective potential risk factors related with TB incidence and drug-related side-effects.

## Results

### Study participants

As a pre-enrolment screening for the RCT, 44,500 rural residents were recruited, 830 of whom were excluded (decline to undergo chest radiography screening (*n* = 506), without questionnaire results (*n* = 135), with history of anti-TB treatment (*n* = 133), and with duplicate results (*n* = 56)), and 43,670 were potentially eligible with completed results of questionnaires survey and chest DR examination. A total of 40,682 participants were further excluded from the second-round screening survey due to normal chest DR examination findings (*n* = 40,451), suspected TB (*n* = 119), and other pulmonary disorders (*n* = 112). The rest 2,988 (6.84%) participants were identified with radiographically inactive TB lesions and all of them were examined by QFT testing. Among them, 855 (28.61%, 855/2,988) participants were identified to be QFT-positive, 2121(70.99%, 2,121/2,988) were QFT-negative and 12 (0.40%, 12/2,988) were indeterminate. Subsequently, the 855 QFT-positive participants with radiographically inactive TB were invited for the second-round screening survey, and 830 of them signed the informed consent form for the RCT if checked to be eligible. Among them, 153 were further excluded due to an ALT and/or AST result >2 ULN (*n* = 69), missing WBC results (*n* = 53), missing ALT and/or AST results (*n* = 11), HCV-positive results (*n* = 9), WBC <2.0 × 10^9^ L^−1^ (*n* = 2), and chest DR results that were inconsistent with the first-round image readings (which were recognized as suspected TB in the second-round screening survey) (*n* = 9). None of the participants had ever received LTBI treatment before, and none was HIV-positive. Finally, 677 participants were identified to be eligible for the RCT and were randomly classified into two groups: 345 in the preventive treatment group and 332 in the control group without treatment (Figure 1).

Detailed characteristics of the participants were shown in Table 1 and Supplementary Table S3. Among the total screened participants, 39.32% (17,172/43,670) were males, and the median age was 59 years. The prevalence of fibrotic lesions was 2.81% (1,230/43,670) and accounted for 41.16% (1,230/2,988) among any radiographically inactive TB lesions. For those with fibrotic lesions, QFT positivity was 25.45% (313/1,230). After randomization, all the variables were evenly distributed between the groups. The distribution of age, baseline IFN-γ level, and active TB by fibrosis size were shown in Supplementary Table S4.

**Table 1.** Characteristics of the study participants included in the intention-to-treat analysis.

Characteristic	Preventive treatment group (*N* = 345)	Untreated control group (*N* = 332)	*P* for *χ*^2^ test
Male	199 (57.68)	194 (58.43)	.843
Age (years)			
Median (Q25–Q75)	63 (54–68)	64 (55–69)	.309
<50	45 (13.04)	40 (12.05)	.924
50–59	90 (26.09)	85 (25.60)	
60–69	140 (40.58)	133 (40.06)	
70–75	70 (20.29)	74 (22.29)	
Body mass index (BMI, kg m^−2^)			
<18.5	14 (4.06)	10 (3.01)	.855
18.5–<24.0	133 (38.55)	124 (37.35)	
24.0–<28.0	134 (50.38)	132 (39.76)	
≥28.0	64 (18.55)	66 (19.88)	
Completed primary school	133 (38.55)	116 (34.94)	.330
Married	309 (89.57)	301 (90.66)	.633
Household per capita income > RMB 2500 yuan	153 (44.35)	157 (47.29)	.443
Ever-smoker	114 (33.04)	103 (31.02)	.574
Current alcohol drinker	86 (24.93)	96 (28.92)	.242
Close contact with a patient with active TB	9 (2.61)	7 (2.11)	.669
History of type II diabetes*	19 (.51)	19 (5.72)	.903
HBsAg positive	7 (2.03)	4 (1.20)	.397^§^
Self-reported history of silicosis	0 (0)	1 (0.30)	.490^§^
Inactive lesions identified by chest radiograph^#^			
With fibrotic lesions	129 (19.05)	114 (16.84)	.459
With nodules	25 (3.69)	23 (3.40)	.872
With calcification	195 (28.80)	187 (27.62)	.959
With pleural incrassation	25 (3.69)	34 (9.94)	.167
Size of the largest fibrosis lesion (mm)^†^			
<20	98 (75.97)	82 (71.93)	.473
≥20	31 (24.03)	32 (28.07)	
Concomitant drugs during treatment^‡^			
Yes	84 (24.35)	–	–
No	261 (75.65)	–	–

Data are presented as *n* (%). Abbreviation: Q25, quartile 25; Q75, quartile 75; TB, tuberculosis. ^‡^Data on taking concomitant drugs were monitored and collected during clinic visits for study drug treatment; therefore, related information on concomitant drug use in untreated control group were not collected. ^#^Every type of radiographically inactive lesions could include only a single lesion or one accompanied with other radiographically inactive lesions. Therefore, the sum of the sample sizes for inactive lesions identified by chest radiographic findings might not be equal to the total of 677. *History of self-reported type II diabetes or fasting blood glucose ≥7 mmol L^−1^. ^†^Classified by Q75 value. ^§^Fisher’s exact test.

### Completion rate of treatment

As shown in Table 2, the rate of treatment completion was 80.00% (276/345), with discontinuing treatment due to refusal to continue (31.88%, 22/69), underlying disease management without hospitalization (27.54%, 19/69), hospitalization due to an underlying disease (15.94%, 11/69), drug side-effects (14.49%, 10/69), unreachable (7.25%, 5/69), and other reasons (2.90%, 2/69), respectively. Details on therapy completion were presented in Supplementary Table S5.

**Table 2.** Completion of the treatment regimens and attributions for treatment discontinuation.

Variables	*n* (%)
Number of completed doses	
11–12 doses (completed)*	276 (80.00)
<11 doses (uncompleted)	69 (20.00)
Attribution for treatment discontinuation	
Unreachable	5 (7.25)
Adverse drug effect	10 (14.49)
Refusal to continue	22 (31.88)
Hospitalization due to underlying disease^†^	11 (15.94)
Management required for underlying disease but without hospitalization^†^	19 (27.54)
Other reasons including right arm trauma and traffic accident	2 (2.90)

*Defined as completed ≥90% doses (i.e. ≥11 doses) of drug therapy. ^†^Underlying disease management included management of hypertension and high fasting blood glucose.

### Protective effect of the treatment regimen

In total, 15 TB cases (nine were microbiologically confirmed and six were clinically diagnosed) were identified during follow-up, eight in the preventive treatment group and seven in the untreated controls (Table 3 and Supplementary Table S6). Supplementary Figure S1 shows the Kaplan–Meier curve of the time to active TB according to the groups. In the ITT analysis (Table 3), the cumulative incidence of total active TB was 2.32% (95% CI: 0.73–3.19%) in the preventive treatment group and 2.11% (95% CI: 0.56–3.65%) in the untreated control group, respectively (*p *= .531). The results did not remarkably change when including only microbiologically confirmed TB cases. The cumulative incidence rate of microbiologically confirmed active TB during a two-year follow-up was 1.16 (95% CI: 0.03–2.29) in the preventive treatment group and 1.51 (95% CI: 0.20–2.82) in the control group (*p *= .485). In the per-protocol analysis (Table 3), the cumulative incidence of microbiologically confirmed TB was 1.09% (95% CI: 0.22–3.14%) in the preventive treatment group. No statistical significance was found when compared with the untreated controls (*p *= .467) as well. Further subgroup analyses showed that the respective protective rate was 55.42% (95% CI: 10.33–93.07%) (*p *= .454) and 80.17% (95% CI: 25.36–97.96%) (*p *= .104) for the participants with fibrosis and for those aged older than 60 years, although it did not reach statistical significance (Table 3). There were no significant differences in the TB incidence by the type of radiographically inactive TB lesions (Supplementary Table S7).

**Table 3.** Incidence of active tuberculosis in the study groups.

	Preventive treatment group		Untreated control group		Difference in cumulative rate^‡^	*P*-value for difference in cumulative rate^¶^	Protective rate* % (95% CI)	Protective rate^†^ % (95% CI)
	Cumulative incidence, n/N (%, 95% CI)	Incidence rate per 100 person-years (95% CI)	Cumulative incidence, n/N (%, 95% CI)	Incidence rate per 100 person-years (95% CI)				
*ITT analysis*								
Microbiologically confirmed and clinically diagnosed cases among total population	8/345 (2.32, 0.73–3.91)	8/650 (1.23, 0.62–2.41)	7/332 (2.11, 0.56–3.65)	7/620 (1.13, 0.55–2.31)	0.21	.531	–	–
Microbiologically confirmed among total population	4/345 (1.16, 0.03––2.29)	4/650 (0.62, 0.24–1.58)	5/332 (1.51, 0.20–2.82)	5/620 (0.81, 0.35–1.88)	−0.35	.485	23.18 (1.83–83.04)	23.46 (1.07–89.69)
Microbiologically confirmed cases among individuals with fibrosis	1/129 (0.78, 0.02–4.24)	1/243 (0.41, 0.07–2.29)	2/114 (1.75, 0.21–6.19)	2/218 (0.92, 0.25–3.29)	−0.97	.454	55.42 (10.33–93.07)	55.43 (6.19–95.91)
Microbiologically confirmed cases among individuals with fibrosis size ≥ 20 mm	0/31 (0)	0/59 (0)	1/32 (3.13, 0.08–9.15)	2/62 (3.23, 0.89–11.03)	−3.13	.508	100.00 (0–55.10)	100.00 (0–54.32)
Microbiologically confirmed cases among individuals aged ≥ 60 years	1/210 (0.48, 0.01–2.62)	1/397 (0.25, 0.04–1.41)	5/207 (2.42, 0.32–4.51)	5/388 (1.29, 0.55–2.98)	−1.94	.104	80.17 (25.36–97.96)	80.62 (16.53–98.87)
*PP analysis*								
Microbiologically confirmed and clinically diagnosed cases among total population	6/276 (2.17, 0.45–3.89)	6/524 (1.15, 0.53–2.48)	7/332 (2.11, 0.56–3.65)	7/620 (1.13, 0.55–2.31)	0.06	.586	–	–
Microbiologically confirmed among total population	3/276 (1.09, 0.22–3.14)	3/524 (0.57, 0.19–1.67)	5/332 (1.51, 0.20–2.82)	5/620 (0.81, 0.35–1.88)	−0.42	.467	27.81 (2.57–84.91)	29.63 (1.68–91.23)
Microbiologically confirmed cases among individuals with fibrosis	1/108 (0.93, 0.02–5.05)	1/206 (0.49, 0.03–3.10)	2/114 (1.75, 0.21–6.19)	2/218 (0.92, 0.25–3.29)	−0.82	.520	46.86 (7.60–90.43)	46.74 (4.48–94.26)
Microbiologically confirmed cases among individuals with fibrosis size ≥ 20 mm	0/27 (0)	0/52 (0)	1/32 (3.13, 0.08–9.15)	2/62 (3.23, 0.89–11.03)	−3.13	.542	100.00 (0–55.10)	100.00 (0–54.32)
Microbiologically confirmed cases among individuals aged ≥ 60 years	1/165 (0.61, 0.02–3.33)	1/316 (0.32, 0.06–1.78)	5/207 (2.42, 0.32–4.51)	5/388 (1.29, 0.55–2.98)	−1.81	.170	74.79 (22.33–96.84)	75.19 (14.48–98.19)

Abbreviation: CI, confidence interval; ITT, intention-to-treat analysis; PP, per-protocol analysis. *Protective rate was calculated by using cumulative incidence. ^†^Protective rate was calculated by using incidence rate per 100 person-years. ^‡^The difference was the rate in the preventive treatment group minus the rate in the untreated control group. ^¶^One-sided *p*-value for Chi-squared test or Fisher’s exact test.

### Adverse events

For 12 deaths that occurred in the two groups during the study period (five in the preventive treatment group and seven in the untreated control group), none of them were attributed to the LTBI treatment (Supplementary Table S8). As shown in Table 4, the proportions of total and serious AEs were 21.45% (74/345) and 2.37% (10/345), respectively. Among 41 (11.88%) participants with drug-related side-effects, the proportions of gastrointestinal reactions (6.38%), neurological symptoms (2.90%), hypersensitivity or allergy (1.74%), influenza-like symptoms (1.45%), and hepatotoxicity (0.58%) were observed. Detailed drug-related side-effects for each dose were shown in Supplementary Table S9. Only one treated case (0.29%) was in hospitalization due to the side-effects. Two participants (0.58%) were identified with elevated ALT/AST higher than normal level, which resolved to the normal level after the administration of the medications had ceased. None of the participants had >3×ULN or >5×ULN, and no renal dysfunction was identified.

**Table 4.** Characteristics of the participants with adverse events and study drug side-effects.

Variables	*n* (%)
Participants	345
Adverse events	
Participants with any of the adverse events	74 (21.45)
Death	0 (0)
Occurrence of serious adverse events	
Yes	10 (2.37)
No	64 (18.55)
Frequency per person	
1 event	53 (15.36)
>1 event	21 (6.07)
Attributed to drug	
Yes	41 (11.88)
No	33 (9.57)
Classified by event severity grade	
Grade 1	56 (16.23)
Grade 2	15 (4.35)
Grade 3	2 (0.58)
Grade 4	1 (0.29)
Side-effects	
Participants with any of the side-effects	41 (11.88)
Specific side-effect	
Gastrointestinal reaction	22 (6.38)
Hyposensitivity or allergy	6 (1.74)
Influenza-like symptoms	5 (1.45)
Neurological symptoms	10 (2.90)
Hepatotoxicity	2 (0.58)
Other drug reactions	3 (0.87)
Categories occurring per person	
1	34 (9.86)
2	5 (1.45)
3	2 (0.58)
Hospitalization due to side-effects	1 (0.29)

### Risk factors

As shown in Supplementary Table S10, univariate analysis showed that no considered variables were statistically significantly associated with the incidence of active TB.

Detailed information on concomitant medication use during preventive treatment was showed in Supplementary Table S11. The frequency of side-effects occurrence among treated participants with concomitant medications was significantly higher than that of those without concomitant medications (27.38% vs. 6.90%, *p* < .001). Multiple logistic regression analysis indicated that the risk of side-effects increased among the participants with concomitant medication (adjusted odds ratio [OR]: 3.41, 95% CI: 2.58–4.68) (Supplementary Table S12).

## Discussion

To the best of our knowledge, this is the first RCT on LTBI testing and treatment in populations with radiographically inactive TB and without history of TB treatment in rural China. This study showed a high burden of radiographically inactive TB among the study participants (6.84%, 2,988/43,670), and the prevalence of LTBI was as high as 28.61% (855/2,988) among these participants. Unfortunately, the studied six-week regimen for LTBI treatment did not show significant protective effect in the total study population. Subsequent exploratory subgroup analyses observed a positive protective effect among the participants with fibrotic lesions (55.42%) and among those aged ≥60 years (80.17%), but it did not reach statistical significance.

Our results suggested that there was a certain proportion of the general population in rural China who might have been infected with MTB and left inactive TB lesions after self-cured. Additionally, the prevalence of LTBI was much higher among individuals with radiographically inactive TB (28.61%, 855/2,988) than that among the general rural adults from the same study site (19.0%, 801/4,223) [15]. Therefore, for the first time as we know, our results disclosed a high burden of radiographically inactive TB with LTBI in rural China. It was essential to explore suitable preventive treatment tools for such a key population. TB preventive treatment studies have been conducted since the 1950s [16,17], and evidences [14,18–20] and recommendations from the WHO [3,21,22] supported its effectiveness for several populations, including for individuals with inactive TB [23–25]. However, the main recommended option for preventive treatment was monotherapy with daily doses of isoniazid for at least six months. In this study, we tried to use a newly developed six-week regimen for this population to improve compliance and feasibility of the preventive treatment. However, we did not observe a significantly positive protective effect for this short-course regimen as observed in our previous studies [8,9]. The finding might be explained by two potential reasons. First, the definition of inactive TB at enrolment in our study was based on four typically radiographical inactive TB lesions adopted by Chinese industrial standard [13]. However, the ability of such a definition based on radiographic abnormalities to specifically represent an inactive TB need to be further explored, as other lung infections may leave similar lesions. Therefore, the potential misclassification may have led to the inclusion of study participants who were not the actual high-risk participants with poor response to the preventive treatment. In the control group, only 1.51% study participants developed microbiologically confirmed TB during two-year follow-up, the unexpected lower incidence of active TB among the control group might support this speculation. In addition, we could not rule out the possibility that our included participants might not be recently infected, as it is commonly observed that half of the lifetime risk of active TB was occurred in the first two years after infection [3]. All in all, the unexpected lower incidence of active TB also limited the power of this study to identify a significant protective effect in the study population. Second, we could not exclude the possibility that the studied short-course regimen was not effective in the population with inactive TB. After all, this study was the first time to practice a short-course regimen in this population worldwide. However, focusing on the study participants with fibrotic lesions, especially those with a fibrotic size ≥20 mm and elderly (≥60 years), a much better protective rate was observed. Although not reaching statistical significance, it indicated that the six-week short-course regimen might produce a more positive effect if we could define the target population with radiographically inactive TB more precisely. Therefore, further studies are urgently needed to improve the definition of inactive TB with comprehensive consideration of the type of radiographic lesions, current MTB infection status, and history of MTB exposure and anti-TB treatment. Such improvement would be crucial for evaluating and expanding LTBI treatment among individuals with radiographically inactive TB in the future.

To improve the guarantee on safety, we adapted the frequency of active side-effects monitoring by revising it from once monthly to twice monthly based on previous research. Therefore, some adverse reactions were handled in time at the initial stage. On the other hand, the participants also received more care, which improved their compliance. During the intervention implementation, only two participants showed moderate liver dysfunction, which was significantly lower than the findings in our previous trial that performed among middle-aged and elderly individuals with LTBI (1.17%) [8]. Increasing the monitoring frequency of adverse reactions will increase the intervention cost, therefore, further studies are needed to balance the risks and benefits of preventive treatment more systematically according to the characteristics of the intervention target population.

Consistent with the recommendation by guidelines from America and Canada, we excluded participants with anti-TB treatment history because there was insufficient evidence to include them as preventive treatment targets. However, some evidence still suggested that this population was at a higher risk of developing disease in high-burden settings [6,7,26]. Therefore, LTBI management for this population, especially those who have been exposed, should not be neglected. In addition, China has a high burden of HBV infection, therefore, participants with HBV were not excluded from this study to prompt individuals co-infected with MTB and HBV to benefit from preventive treatment in future [27]. However, the epidemic level of HCV was relatively low in China [28] and HCV infection was associated with anti-TB treatment-induced hepatotoxicity [29–31]. Therefore, given safety concerns regarding potentially hepatotoxicity, participants with HCV were excluded from this study. Regarding participants with HIV infection, the reason for their exclusion was because the prevalence was relatively low in China [32] and there was lacking evidence of the safety and efficacy of the six-week regimen among immunodeficient populations. Therefore, exploratory and confirmatory research should be conducted among these specific populations with a well-conducted RCT design in the future.

Our study has several limitations. First, some TB cases especially subclinical TB cases might have been under-diagnosed because only participants with suspected TB were transferred for further microbiological tests. The unexpected lower incidence of active disease limited us to detect expected treatment effects and get a conclusive finding, considering the current sample size. Although not reaching statistical significance, a positive protective effect was observed among the subgroups with fibrotic lesion and among those aged ≥60 years, further studies with sufficient outcome events from larger sample size are needed to verify our results as calculated in Supplementary Table S13. Second, participants in this trial were not blinded because an untreated control group was used. To reduce the risk of potential bias, the panel members for evaluation and diagnosis of the active TB were fully unaware of the trial-group assignments. In addition, AE evaluation was mainly based on laboratory measurements and graded using a standardized, widely used CTC-AE. Third, as there is no gold standard for LTBI testing, infection status and time of infection acquisition were not known with certainty. There is a need to further explore whether these factors would influence treatment effectiveness.

In conclusion, a high burden of radiographically inactive TB without anti-TB treatment history was identified in rural China. No significant efficacy of the six-week regimen was observed for TB preventive treatment among this key population, which might have been influenced by ages and the type of radiological lesion. A more precise definition of radiographically inactive TB is needed to verify our preliminary findings and to explore precise context-specific intervention for this population in future.

## Supplementary Material

Unmarked_up_Supplementary_materials_R1.docx

## Data Availability

This study is registered at www.chictr.org.cn with identifier ChiCTR-1800018224. The corresponding author can provide, upon request, individual participant data that underlie the results reported in this article after applying necessary measures to guarantee that no individual is identified or identifiable.
