# Supplementary material for: Tuberculosis preventive treatment among individuals with inactive tuberculosis suggested by untreated radiographic abnormalities: a community-based randomized controlled trial
Source: Emerg Microbes Infect. 2023 Jan 27;12(1):e2169195. doi: 10.1080/22221751.2023.2169195 (PMC9888474; doi:10.1080/22221751.2023.2169195)
Supplement: Unmarked_up_Supplementary_materials_R1.docx [file TEMI_A_2169195_SM0047.docx]

**Supplementary Table 1 Standard report form of chest digital radiography**

- 1. No abnormality is found

| - 2. Abnormal: suspected active pulmonary tuberculosis (TB) lesions - Lesions site - Whether there is cavity: □2-1) yes; □2-2) no. - 3. Abnormal: radiographically inactive pulmonary TB lesions - Lesions site - Types of lesions (multiple choices are allowed):   □3-1) Fibrotic lesions (with clear edges)  □3-2) Nodules  □3-3) Calcification (isolated or primary)  □3-4) Pleural incrassation |
| --- |

| - 4. Abnormal, non-TB lesions, with the impression of:   Viewer: Date of viewing: |
| --- |

**Supplementary Table 2 Evaluation of the association between adverse events and study drugs ^[1,2]^**

|  | | Time Sequence | Documentary evidence | Relief disappears after withdrawal | Reappear after re-administration | | Confounding factor |
| --- | --- | --- | --- | --- | --- | --- | --- |
| Sure | Yes | | Yes | Yes | Yes | | Cannot explain it |
| Probably | Yes | | Yes | Yes | Never used again | | Cannot explain it |
| Maybe | Yes | | Yes | Yes/No | Never used again | Cannot rule it out | |
| Impossible | None | | Yes/No | None/unknown | Unused/None | | Cannot rule it out |
| To be evaluated | Incomplete information | | | | | | |
| Cannot be evaluate | Serious loss of information | | | | | | |

**Reference**

1. Wang L, Chen M, Wan L, et al. Handbook for the Administration of Anti-tuberculosis Drugs-edition 2. Beijing: People’s Military Medical Press, 2011
2. National Health Commission of the People’s Republic of China. Administrative Measures for Reporting and Monitoring Adverse Drug Reactions (Ministry of Health Decree No. 81). 2011.

**Supplementary Table 3 Major characteristics of the study participants**

| **Variables** | **n** | **(%)** |
| --- | --- | --- |
| **Total** | 43,670 | 100 |
| **Sex** |  |  |
| Male | 17,172 | 39·32 |
| Female | 26,498 | 60·68 |
| **Age** |  |  |
| Median (IQR) | 59 (51-66) |  |
| 18-49 years | 8,081 | 18·50 |
| 50-59 years | 14,476 | 33·15 |
| 60-69 years | 15,212 | 34·83 |
| ≥70 years | 5,901 | 13·51 |
| **Chest radiographic results** |  |  |
| Normal | 40,451 | 92·63 |
| With suspected active TB lesions | 119 | 0·27 |
| With other pulmonary diseases | 112 | 0·26 |
| With any radiographically inactive TB lesion* | 2,988 | 6·84 |
| With fibrosis | 1,230 | 41·16 |
| With calcification | 1,639 | 54·85 |
| With nodule | 216 | 7·23 |
| With pleural incrassation | 176 | 5·89 |
| **IGRA positivity for individuals with** **radiographically inactive TB lesion*** |  |  |
| With fibrosis | 313 | 25·45 |
| With calcification | 493 | 30·08 |
| With nodule | 61 | 28·24 |
| With pleural incrassation | 71 | 40·34 |

Abbreviation: IGRA, Interferon Gamma Release Assay. IQR, interquartile range. TB, tuberculosis.

*Chest radiographic lesions of prior TB (i.e., fibrosis, calcification, nodule, and pleural incrassation) were identified by an expert panel. Some participants might have had multiple types of lesions; the sum reported was not equal to the total.

**Supplementary Table 4 Distribution of age, baseline IFN-γ level and** **microbiologically confirmed tuberculosis by fibrosis size**

| **Characteristic** | **Size of fibrosis**  **< 20 mm** | **Size of fibrosis**  **≥ 20 mm** | ***P* for χ^2^ test** |
| --- | --- | --- | --- |
| **Age (years)** |  |  |  |
| < 60 | 62 (34·44) | 22 (34·92) | 0·946 |
| ≥ 60 | 118 (65·56) | 41 (65·08) |  |
| **Baseline median level of IFN-γ (IU/mL)** |  |  |  |
| < 1·80 | 90 (50·00) | 36 (57·14) | 0·329 |
| ≥ 1·80 | 90 (50·00) | 27 (42·86) |  |
| **Occurrence of microbiologically confirmed tuberculosis** |  |  |  |
| Yes | 3 (1·67) | 3 (4·76) | 0·256^§^ |
| No | 177 (98·33) | 60 (95·24) |  |

^§^ Fisher’s exact test.

**Supplementary Table 5 Completed doses of the 6-week intervention regimen**

| **Completed doses** | **Intervention regimen ^#^**  **n (%)** |
| --- | --- |
| **0 dose** | 12 (3·48) ^*^ |
| **1 dose** | 7 (2·03) |
| **2 doses** | 6 (1·74) |
| **3 doses** | 3 (0·87) |
| **4 doses** | 4 (1·16) |
| **5 doses** | 8 (2·32) |
| **6 doses** | 8 (2·32) |
| **7 doses** | 6 (1·74) |
| **8 doses** | 5 (1·45) |
| **9 doses** | 4 (1·16) |
| **10 doses** | 6 (1·74) |
| **11 doses** | 12 (3·48) |
| **12 doses** | 264 (76·52) |

^#^ The intervention group completed six weeks of twice-weekly rifapentine plus isoniazid (both with a maximum dose of 600 mg).

^*^ Five participants were unreachable and seven refused to continue.

**Supplementary Table 6 Characteristics of the identified patients with incident tuberculosis**

| **ID** | **Sex** | **Age** | **Group** | **Countryside site** | **Time of**  **diagnosis** | **Smear** | **Culture** | **GeneXpert** | **Diagnosis** | **Rifampicin resistance by** **GeneXpert** |
| --- | --- | --- | --- | --- | --- | --- | --- | --- | --- | --- |
| **1** | Male | 58 | Preventive treatment group | Mengzhuang | 30-Dec-20 | Negative | Negative | Positive | Microbiologically confirmed | Negative |
| **2** | Female | 59 | Preventive treatment group | Nianluo | 1-Aug-20 | Negative | Positive | Positive | Microbiologically confirmed | Negative |
| **3** | Male | 74 | Preventive treatment group | Mazhuang | 30-Dec-19 | Negative | Positive | Positive | Microbiologically confirmed | Negative |
| **4** | Female | 54 | Preventive treatment group | Bafuzhao | 1-Aug-20 | Negative | Positive | Negative | Microbiologically confirmed | Negative |
| **5** | Male | 69 | Untreated control group | Shijia | 1-Aug-20 | Negative | Positive | Positive | Microbiologically confirmed | Negative |
| **6** | Female | 73 | Untreated control group | Chepengzhang | 1-Aug-20 | Negative | Positive | Positive | Microbiologically confirmed | Negative |
| **7** | Male | 73 | Untreated control group | Leijia | 1-Aug-20 | Positive | Positive | Positive | Microbiologically confirmed | Negative |
| **8** | Male | 69 | Untreated control group | Ranjia | 1-Aug-20 | Negative | Positive | Positive | Microbiologically confirmed | Negative |
| **9**^#^ | Female | 69 | Untreated control group | Rongzhuang | 24-Aug-20 | Negative | Positive | Positive | Microbiologically confirmed | Negative |
| **10** | Female | 49 | Preventive treatment group | Cangzhai | 30-Dec-19 | Negative | Negative | Negative | Clinically diagnosed | Negative |
| **11** | Female | 73 | Preventive treatment group | Wansheng | 30-Dec-19 | Negative | Negative | Negative | Clinically diagnosed | Negative |
| **12** | Male | 65 | Untreated control group | Nanren | 30-Dec-19 | Negative | Negative | Negative | Clinically diagnosed | Negative |
| **13** | Male | 67 | Preventive treatment group | Gouzhang | 30-Dec-19 | Negative | Negative | Negative | Clinically diagnosed | Negative |
| **14** | Male | 59 | Untreated control group | Shapochi | 15-Sep-19 | Negative | Negative | Negative | Clinically diagnosed | Negative |
| **15** | Male | 61 | Preventive treatment group | Ranjia | 30-Dec-19 | Negative | Negative | Negative | Clinically diagnosed | Negative |

^#^ Registered in the national Tuberculosis Information Management System during the follow-up period.

**Supplementary Table 7 Incidence of the identified** **patients with incident tuberculosis by the type of radiographically inactive TB lesions**

| **Types of radiographically inactive TB lesions** ^§^ | **Preventive treatment group** | **Untreated control group** | ***P* for Fisher’s exact test** |
| --- | --- | --- | --- |
| **Microbiologically confirmed and clinically diagnosed cases** |  |  |  |
| With fibrotic lesions | 4/129 (3.10) | 2/114 (1.75) | 0.492 |
| With nodules | 0/25 (0) | 0/23 (0) | - |
| With calcification | 6/195 (3.08) | 4/187 (2.14) | 0.566 |
| With pleural incrassation | 0/25 (0) | 1/34 (2.94) | 0.576 |
| **Microbiologically confirmed cases** |  |  |  |
| With fibrotic lesions | 1/129 (0.78) | 2/114 (1·75) | 0.454 |
| With nodules | 0/25 (0) | 0/23 (0) | - |
| With calcification | 3/195 (1.54) | 2/187 (1.70) | 0.321 |
| With pleural incrassation | 0/25 (0) | 1/34 (2.94) | 0.576 |

^§^ Every type of radiographically inactive lesions may include only a single lesion and may be accompanied by other radiographically inactive lesions. Therefore, the sum of the sample sizes for inactive lesions on chest radiographs may not be equal to the total.

**Supplementary Table 8 Characteristics of patients who died during the study period**

| No. | **ID** | **Age (years)** | **Sex** | **Group** | **Completed doses** | **Date of death** | **Reasons for death** |
| --- | --- | --- | --- | --- | --- | --- | --- |
| 1 | 20400442 | 67 | Male | Preventive treatment group | 12 | 3- February-2020 | Cerebral infarction |
| 2 | 20503972 | 56 | Male | Preventive treatment group | 12 | 17-September-2019 | Lung cancer |
| 3 | 20700480 | 74 | Male | Preventive treatment group | 11 | 26-January-2019 | Chronic obstructive pulmonary disease |
| 4 | 21001354 | 71 | Female | Preventive treatment group | 12 | 15-December-2018 | Lung cancer |
| 5 | 21502556 | 72 | Male | Preventive treatment group | 12 | 23-April-2020 | Traffic accident |
| 6 | 20400927 | 65 | Female | Untreated control group | 0 | 18-April-2020 | Colorectal carcinoma |
| 7 | 20507969 | 66 | Male | Untreated control group | 0 | 5- November-2020 | Emphysema |
| 8 | 20700283 | 70 | Male | Untreated control group | 0 | 1-August-2020 | Cerebral infarction |
| 9 | 20701083 | 72 | Male | Untreated control group | 0 | 19-January-2020 | Acute myocardial infarction |
| 10 | 20801158 | 73 | Male | Untreated control group | 0 | 27-June-2019 | Acute myocardial infarction |
| 11 | 20801166 | 71 | Male | Untreated control group | 0 | 5-January-2020 | Acute myocardial infarction |
| 12 | 21201120 | 63 | Female | Untreated control group | 0 | 8-December-2020 | Interstitial lung disease |

**Supplementary Table 9 Proportion of drug-related side effects other than liver injury**

|  | **Dose 1** | **Dose 2** | **Dose 3** | **Dose 4** | **Dose 5** | **Dose 6** | **Dose 7** | **Dose 8** | **Dose 9** | **Dose 10** | **Dose 11** | **Dose 12** |
| --- | --- | --- | --- | --- | --- | --- | --- | --- | --- | --- | --- | --- |
| **Gastrointestinal reaction** | 3 (0·87) | 10 (2·90) | 5 (1·45) | 2 (0·58) | 7 (2·03) | 5 (1·45) | 2 (0·58) | 2 (0·58) | 2 (0·58) | 1 (0·29) | 1 (0·29) | 1 (0·29) |
| **Neurological symptoms** | 2 (0·58) | 2 (0·58) | 1 (0·29) | 1 (0·29) | 1 (0·29) | - | 2 (0·58) | 2 (0·58) | 1 (0·29) | - | - | - |
| **Hyposensitivity or allergy** | 2 (0·58) | 1 (0·29) | - | 1 (0·29) | - | - | 1 (0·29) | - | - | 2 (0·58) | - | - |
| **Influenza-like symptoms** | - | - | - | - | - | 2 (0·58) | 1 (0·29) | - | - | 2 (0·58) | - | - |
| **Other drug reactions** | - | 1 (0·29) | - | - | - | - |  | - | - | - | - | - |

**Supplementary Table 10 Risk factors related to the incidence of microbiologically confirmed active TB**

| **Variables** | **n/N (%)** | **Univariable Cox regression** | |
| --- | --- | --- | --- |
|  |  | **Hazard Ratio (95%CI)** | ***P* value** |
| **Group** |  |  |  |
| Untreated controls | 5/332 (1.51) | Reference |  |
| Preventive treatment group | 4/345 (1.16) | 0.93 (0.34-2.56) | 0.886 |
| **Age (years)** |  |  |  |
| < 60 | 3/260 (1.15) | Reference |  |
| ≥ 60 | 6/417 (1.44) | 1.24 (0.42-3.62) | 0.699 |
| **Sex** |  |  |  |
| Female | 4/284 (1.41) | Reference |  |
| Male | 5/393 (1.27) | 1.05 (0.37-2.94) | 0.929 |
| **BMI (Kg/m^2^)** |  |  |  |
| <18.5 | 9/677 (1.33) | **-** |  |
| 18.5-<24.0 | - | - |  |
| 24.0-<28.0 | - | - |  |
| ≥ 28.0 | - | - |  |
| **Ever smoked** |  |  |  |
| No | 6/460 (1.30) | Reference |  |
| Yes | 3/217 (1.38) | 1.41 (0.50-3.96) | 0.514 |
| **Current alcohol drinking** |  |  |  |
| No | 8/495 (1.62) | Reference |  |
| Yes | 1/182 (0.55) | 0.41 (0.09-1.80) | 0.234 |
| **Close contact with a patient with active TB** |  |  |  |
| No | 9/661 (1.36) | Reference |  |
| Yes | 0/16 (0) | - | 0.991 |
| **With a history of T2DM**^*^ |  |  |  |
| No | 9/639 (1.41) | Reference |  |
| Yes | 0/38 (0) | - | 0.990 |
| **HBsAg** |  |  |  |
| Negative | 9/666 (1.35) | Reference |  |
| Positive | 0/11 (0) | - | 0.991 |
| **Types of radiographically inactive lesions** |  |  |  |
| With fibrotic lesions | 3/243 (1.23) | - |  |
| With nodules | 0/48 (0) | - |  |
| With calcification | 5/382 (1.31) | - |  |
| With pleural incrassation | 1/59 (1.69) | - |  |

BMI, body mass index; CI, confidence interval; OR, odds ratio; T2DM, type II diabetes mellitus; TB, tuberculosis.

^*^ History of self-report type II diabetes or fasting blood glucose ≥ 7 mmol.L^−1^. ^§^ Fisher’s exact test

**Supplementary Table 11 Details of concomitant drugs during preventive treatment**

| **ID** | **Sex** | **Age (years)** | **Concomitant medication** |
| --- | --- | --- | --- |
| 1 | Male | 71 | Amlodipine besylate tablets, acetylspiramycin tablets, prednisone acetate tablets, diclofenac sodium enteric-coated tablets, reserpine tablets |
| 2 | Female | 65 | Compound Danshen tablets*, aspirin enteric-coated tablets |
| 3 | Female | 59 | Aspirin tablets, simvastatin |
| 4 | Male | 63 | Prednisone, ibuprofen |
| 5 | Male | 64 | Qianliekang tablet*, amoxicillin granules |
| 6 | Male | 66 | Zhonghua Jiangtang Capsule* |
| 7 | Male | 63 | Indapamide, simvastatin |
| 8 | Female | 64 | Losartan, amlodipine |
| 9 | Female | 55 | Sartan, indapamide |
| 10 | Female | 67 | Captopril, simvastatin |
| 11 | Male | 53 | Metoprolol succinate sustained-release tablets, atorvastatin calcium |
| 12 | Female | 74 | Indapamide tablets |
| 13 | Female | 61 | Compound reserpine tablet*, rosuvastatin tablet, Xueshuantong*, Yangxueqingnao granule*, nimesulide |
| 14 | Male | 69 | Hydrochlorothiazide tablets |
| 15 | Male | 64 | Indapamide tablets |
| 16 | Female | 52 | nifedipine |
| 17 | Male | 74 | Compound reserpine tablets* |
| 18 | Female | 71 | Simvastatin capsules, nifedipine sustained-release tablets, compound reserpine tablets*, Liuweidihuang pills*, gliclazide tablets |
| 19 | Male | 75 | Compound reserpine tablets* |
| 20 | Female | 67 | Nifedipine sustained-release tablets |
| 21 | Female | 49 | Enteric aspirin, simvastatin |
| 22 | Male | 56 | Nifedipine |
| 23 | Female | 65 | Indapamide |
| 24 | Female | 74 | Available Kyushin Pills* |
| 25 | Male | 70 | Captopril, reserpine |
| 26 | Female | 73 | Metoprolol, valsartan |
| 27 | Female | 47 | Metformin tablets, glimepiride tablets |
| 28 | Male | 73 | Metformin tablets, gliclazide tablets |
| 29 | Female | 74 | Dizziness*, neck rehabilitation* |
| 30 | Male | 53 | Niuhuang Jiedu tablets* |
| 31 | Female | 56 | Metformin tablets, compound danshen injection* |
| 32 | Male | 60 | Aspirin enteric-coated tablets, rosuvastatin calcium tablets, metformin hydrochloride enteric-coated tablets, pioglitazone hydrochloride and glimepiride dispersible tablets. |
| 33 | Female | 69 | Amlodipine hydrochloride tablets, aspirin |
| 34 | Female | 54 | melbine |
| 35 | Male | 70 | Gu shi zai zao wan*, bu gu wan* |

**Supplementary Table 11 Details of concomitant drugs during preventive treatment** **(continued)**

| **ID** | **Sex** | **Age (years)** | **Concomitant medication** |
| --- | --- | --- | --- |
| 36 | Male | 52 | Amoxicillin, vitamin B2 |
| 37 | Male | 69 | Nifedipine tablets, compound tetrazine reserpine tablets |
| 38 | Female | 70 | Tongxinluo capsule, betaloc |
| 39 | Female | 51 | Metformin tablets, amlodipine besylate tablets, enalapril, licorice tablets*, amoxicillin capsules, banlangen granules. |
| 40 | Male | 53 | Liuwei Dihuang Pills* |
| 41 | Female | 66 | Aspirin enteric-coated tablets, isosorbide mononitrate tablets, simvastatin tablets, metoprolol tartrate, Beijing Jiangya No.0* |
| 42 | Female | 73 | Ginkgo biloba soft micelles*, yindanxinnaotong soft micelles*, compound chlorzoxazone micelles*, metformin hydrochloride tablets, simvastatin micelles |
| 43 | Male | 68 | Fluvastatin sodium capsules, aspirin. |
| 44 | Female | 73 | Simvastatin capsules, nifedipine controlled-release tablets, valsartan dispersible tablets, gliclazide sustained-release tablets, metformin hydrochloride sustained-release tablets, aspirin enteric-coated tablets |
| 45 | Male | 63 | Annaijin tablets* |
| 46 | Male | 68 | Losartan potassium hydrochlorothiazide tablets, amlodipine besylate tablets |
| 47 | Male | 55 | Nifedipine sustained-release tablets, compound reserpine tablets * |
| 48 | Male | 63 | Compound reserpine tablets* |
| 49 | Male | 61 | Nifedipine sustained-release tablets |
| 50 | Male | 65 | Amoxicillin, yiliketeling*, licorice tablets, analgin, bingduling*, quick-acting cold capsule |
| 51 | Female | 69 | Nifedipine tablets, amoxicillin, licorice tablets, keteling*, bingduling |
| 52 | Female | 74 | Naoxintong*,songlingxuemaikang* |
| 53 | Male | 63 | Metoprolol, nitrendipine |
| 54 | Male | 69 | Nifedipine sustained-release tablets |
| 55 | Female | 61 | Nifedipine, spiramycin tablets, sanqishang tablets*, anluotong* tablets, ibuprofen |
| 56 | Female | 67 | Nifedipine sustained-release tablets |
| 57 | Male | 61 | Atorvastatin, compound Xueshuantong*, Shihuida*, aspirin, metformin |
| 58 | Female | 58 | Reserpine |
| 59 | Female | 57 | Acarbose tablets, gliclazide sustained-release tablets, Maixuekang capsules, aspirin enteric-coated tablets, rosuvastatin calcium, clopidogrel hydrogen sulfate tablets |
| 60 | Male | 58 | Gentamicin procainavir |
| 61 | Female | 69 | Xiaoyan Zhike tablets* |
| 62 | Female | 72 | Metformin sustained-release tablets, gliclazide |
| 63 | Male | 52 | Phenytoinna |
| 64 | Male | 72 | Naoxintong capsule*, losartan potassium hydrochlorothiazide tablets |

**Supplementary Table 11 Details of concomitant drugs during preventive treatment (continued)**

| **ID** | **Sex** | **Age (years)** | **Concomitant medication** |
| --- | --- | --- | --- |
| 65 | Female | 68 | Nifedipine tablets |
| 66 | Male | 71 | Budesonide formoterol powder inhalation, chlorpheniramine |
| 67 | Male | 52 | Nifedipine sustained-release tablets |
| 68 | Male | 64 | Ginkgo honey ring oral solution* |
| 69 | Male | 71 | compound reserpine tablets* |
| 70 | Male | 60 | Aspirin enteric-coated tablets, losartan potassium hydrochlorothiazide tablets |
| 71 | Female | 67 | Metformin hydrochloride tablets, glibenclamide tablets |
| 72 | Male | 53 | Compound reserpine tablets, nifedipine, felodipine sustained-release tablets, atorvastatin calcium, losartan potassium hydrochlorothiazide tablets |
| 73 | Male | 61 | Nano Fuding |
| 74 | Male | 56 | Liuwei Dihuang Pil*l, Zhuanggu Joint Pill* |
| 75 | Male | 74 | Huanglian tablet* |
| 76 | Male | 57 | Hypoglycemic drug |
| 77 | Male | 55 | Jiangtangshu* |
| 78 | Female | 54 | melbine |
| 79 | Female | 71 | Shu Billy, puhaisuo |
| 80 | Female | 62 | Wenxue Granule*, Isosorbide Monoacetate Tablets |
| 81 | Female | 72 | Aspirin enteric-coated tablets and nifedipine sustained-release tablets |
| 82 | Female | 67 | Metformin hydrochloride sustained release tablets |
| 83 | Female | 72 | Roxithromycin, cold tablets |
| 84 | Female | 49 | Xianlinggu capsule*, Xubi tablet* |

* Traditional Chinese Medicine (TCM)

**Supplementary Table 12 Risk factors associated with the occurrence of side-effects**

| **Variables** | **Proportion of side-effects**  **n/N (%)** | ***P* for χ^2^ test** | **Adjusted OR (95%CI)** |
| --- | --- | --- | --- |
| **Age (years)** |  |  |  |
| < 60 | 17/135 (12·59) | 0·744 |  |
| ≥ 60 | 24/210 (11·43) |  |  |
| **Sex** |  |  |  |
| Female | 20/199 (10·05) | 0·219 |  |
| Male | 21/146 (14·38) |  |  |
| **BMI (Kg/m^2^)** |  |  |  |
| < 18·5 | 2/14 (14·29) | 0·489 |  |
| 18·5-<24·0 | 17/133 (12·78) |  |  |
| 24·0-<28·0 | 18/134 (13·43) |  |  |
| ≥ 28·0 | 4/64 (6·25) |  |  |
| **Ever smoked** |  |  |  |
| No | 28/231 (12·12) | 0·846 |  |
| Yes | 13/114 (11·40) |  |  |
| **Current alcohol drinking** |  |  |  |
| No | 33/259 (12·74) | 0·393 |  |
| Yes | 8/86 (9·30) |  |  |
| **With a history of T2DM**^*^ |  |  |  |
| No | 38/326 (11·66) | 0·588 |  |
| Yes | 3/19 (15·79) |  |  |
| **HBsAg** |  |  |  |
| Negative | 41/338 (12·13) | 0·326 ^§^ |  |
| Positive | 0/7 (0) |  |  |
| **Types of radiographically inactive lesions** |  |  |  |
| With fibrotic lesions | 15/129 (11·63) | 0·275 |  |
| With nodules | 3/23 (13·04) |  |  |
| With calcification | 16/170 (9·42) |  |  |
| With pleural incrassation | 7/23 (30·43) |  |  |
| **Concomitant with other drugs** |  |  |  |
| No | 18/261 (6·90) | <0·001 | Ref. |
| Yes | 23/84 (27·38) |  | 3·41 (2·58-4·68) |

BMI, body mass index; CI, confidence interval; OR, odds ratio; T2DM, type II diabetes mellitus.

^*^ History of self-report type II diabetes or fasting blood glucose ≥ 7 mmol.L^−1^.

^§^ Fisher’s exact test.

**Supplementary Table 13 Power of the present study and expected sample size in further study**

| **Subgroups** | **Observations in the present study** | | | **Expected sample size under 80% power in future study** |
| --- | --- | --- | --- | --- |
|  | **Protective effect (%)** | **Incidence of microbiologically confirmed**  **active tuberculosis**  **in untreated controls in 2-year follow-up**  **% (n/N)** | **Power of the study**  **(%)** |  |
| With fibrosis | 55% | 1.75 (2/114) | 9.98 | 4328 |
| Aged ≥ 60 years | 80% | 2.42 (5/207) | 38.21 | 1176 |


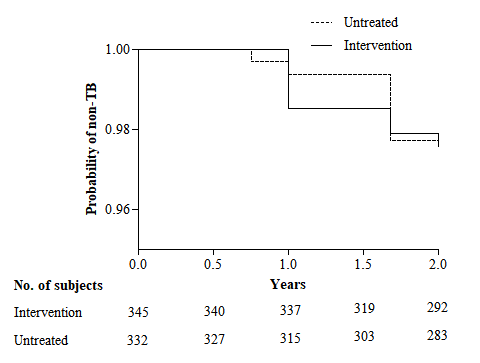


Log-rank

p=0.885

**1A: Intention-to-treat population**

Log-rank

p=0.605


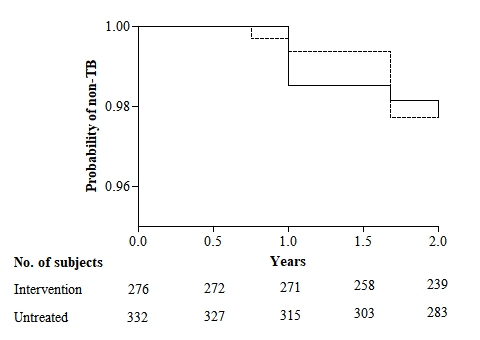


**1B: Per-Protocol population**

**Supplementary Figure 1 Kaplan-Meier curve of time to tuberculosis according to study groups.** 1A: Intention-to-treat population; 1B: Per-protocol population. The number of participants who did not develop the disease were listed according to study groups.

**The LATENTTB TRIAL2-NSTM team member list:**

1. NHC Key Laboratory of Systems Biology of Pathogens, Institute of Pathogen Biology, and Center for Tuberculosis Research, Chinese Academy of Medical Sciences and Peking Union Medical College, Beijing 100730, P.R. China.

Lei Gao, Qi Jin, Haoran Zhang, Henan Xin, Ying Du, Xuefang Cao, Boxuan Feng, Yijun He, Yongpeng He.

1. The Sixth People’s Hospital of Zhengzhou, Zhengzhou 400060, China.

Yu Chen, Wei Cui, Ling Guan, Xueling Guan, Jianmin Liu, Fei Shen.

1. Center for Diseases Control and Prevention of Zhongmu, Zhongmu 451450, China.

Xia Chen, Hairui Chen, Hongbin Guo, Wuying Hao, Zhenwei He, Jie Jiao, Jie Li, Ping Li, Shen Li, Yanfen Li, Zhen Li, Zhenzhen Li , Jingxia Liu, Juntao Liu, Ning Liu, Zisen Liu, Qingtao Lou, Xiaojun Lou, Lu Lu, Shanshan Ma, Wuyi Mao, Shouguo Pan, Yanan Peng, Jianguo Ran, Liujie Shan, Meng Qin, Fei Shang, Xiaoming Song, Li Wan, Liuyan Wan, Dakuan Wang, Jing Wang, Kun Wang(1), Kun Wang(2), Junhong Wei, Fang Wen, Weiling Wu, Shuangqiang Xiao, Hui Xu, Zhigang Xu, Chao Yan, Jiaoxia Yan, Jinfu Yang, Yangtao Yu, Bin Zhang, Lichao Zhang, Yu Zhang, Zhanjiang Zhang, Herong Zhao, Junhui Zheng, Yonghui Zhu.
